# Taurine and Astrocytes: A Homeostatic and Neuroprotective Relationship

**DOI:** 10.3389/fnmol.2022.937789

**Published:** 2022-07-05

**Authors:** Sofía Ramírez-Guerrero, Santiago Guardo-Maya, Germán J. Medina-Rincón, Eduardo E. Orrego-González, Ricardo Cabezas-Pérez, Rodrigo E. González-Reyes

**Affiliations:** ^1^Grupo de Investigación en Neurociencias (NeURos), Centro de Neurociencias Neurovitae-UR, Instituto de Medicina Traslacional (IMT), Escuela de Medicina y Ciencias de la Salud, Universidad del Rosario, Bogotá, Colombia; ^2^Grupo de Investigación en Ciencias Biomédicas GRINCIBIO, Facultad de Medicina, Universidad Antonio Nariño, Bogotá, Colombia

**Keywords:** taurine, astrocytes, hypotaurine, glia, neuron, neuroprotection, brain

## Abstract

Taurine is considered the most abundant free amino acid in the brain. Even though there are endogenous mechanisms for taurine production in neural cells, an exogenous supply of taurine is required to meet physiological needs. Taurine is required for optimal postnatal brain development; however, its brain concentration decreases with age. Synthesis of taurine in the central nervous system (CNS) occurs predominantly in astrocytes. A metabolic coupling between astrocytes and neurons has been reported, in which astrocytes provide neurons with hypotaurine as a substrate for taurine production. Taurine has antioxidative, osmoregulatory, and anti-inflammatory functions, among other cytoprotective properties. Astrocytes release taurine as a gliotransmitter, promoting both extracellular and intracellular effects in neurons. The extracellular effects include binding to neuronal GABA_A_ and glycine receptors, with subsequent cellular hyperpolarization, and attenuation of *N*-methyl-D-aspartic acid (NMDA)-mediated glutamate excitotoxicity. Taurine intracellular effects are directed toward calcium homeostatic pathway, reducing calcium overload and thus preventing excitotoxicity, mitochondrial stress, and apoptosis. However, several physiological aspects of taurine remain unclear, such as the existence or not of a specific taurine receptor. Therefore, further research is needed not only in astrocytes and neurons, but also in other glial cells in order to fully comprehend taurine metabolism and function in the brain. Nonetheless, astrocyte’s role in taurine-induced neuroprotective functions should be considered as a promising therapeutic target of several neuroinflammatory, neurodegenerative and psychiatric diseases in the near future. This review provides an overview of the significant relationship between taurine and astrocytes, as well as its homeostatic and neuroprotective role in the nervous system.

## Introduction

More than 900 natural amino acids are currently known, although only around 2% of them are encoded in the genetic code of eukaryotes and used to synthesize proteins, thereby called “proteinogenic amino acids” (Yamane et al., 2010). The other 98% correspond to “non-proteinogenic” or “non-coded” amino acids, such as taurine, which are not coded into the DNA and are therefore not involved in protein synthesis, but have important physiological functions (Popova and Koksharova, 2016; Fichtner et al., 2017). Taurine is considered the most abundant free amino acid present in the brain, retina, and muscle (Ripps and Shen, 2012). For example, human levels of taurine can range from 1 to 20 μmol/g in the brain, 30 to 40 μmol/g in the retina, and around 50 to 100 μmol/L in plasma (Wójcik et al., 2010).

The word taurine comes from the Latin *taurus*, meaning bull, the species from which it was first isolated (Caspi et al., 2018). Taurine (2-aminoethanesulfonic acid) is a beta-amino acid, with a molecular weight of 125.15 g/mol and a wide distribution in animal tissues (Caspi et al., 2018). It differs from other amino acids, due to the position of its amino group on the beta-carbon and the presence of a sulfonic acid group with a low pKa instead of the conventional carboxylic acid group (Hayes and Sturman, 1981). Taurine is endogenously synthesized in mammalian tissues, especially in the brain, heart, retina, and liver cells as part of the L-cysteine and L-methionine metabolic pathway (Huxtable, 1992). This process requires vitamin B6 as an enzyme cofactor; therefore, its dietary deficiency can lead to taurine depletion (Park and Linkswiler, 1970; Spinneker et al., 2007). Even though there are endogenous mechanisms for taurine production, an exogenous supply of taurine is required to meet physiological needs, especially in infants, in which taurine is a conditionally essential amino acid (Papet et al., 2019; Wu, 2020). Moreover, taurine is highly enriched in meat, seafood, fish, and milk (Froger et al., 2014), and a major component of energy drinks, along with caffeine and B-group vitamins (Piccioni et al., 2021). Taurine uptake by tissues is predominantly mediated by the chloride sodium-dependent taurine transporter (TauT) encoded by the *SLC6A6* gene (Baliou et al., 2020). However, taurine and hypotaurine transport has also been described through the GABA transporter 2 (GAT-2) in the blood-brain barrier (BBB) (Nishimura et al., 2018). Although TauT transporter predominates in the plasma membrane, studies with mouse fibroblasts models have identified its presence in the nucleus (Voss et al., 2004), whereas other models with HeLa cells propose its existence within the mitochondria (Suzuki et al., 2002).

Taurine has diverse functions in the cells, particularly cytoprotective actions through its antioxidative and anti-inflammatory effects (Schaffer and Kim, 2018). This amino acid neutralizes hypochlorous acid through the formation of taurine chloramine, a more stable molecule, and thus, diminishes the generation of reactive oxygen species (ROS) (Weiss et al., 1982). Similarly, taurine conjugates with a tRNA to enhance the expression of the nicotinamide adenine dinucleotide (NAD)-ubiquinone oxidoreductase chain 6, a subunit of the respiratory chain complex I, associated with the reduction of oxidative stress (Jong et al., 2012; Schaffer et al., 2014). Furthermore, taurine deficiency in heart tissue reduces glucose oxidation due to a decrease in pyruvate followed by an increase in lactate (Schaffer et al., 2016). Such a rise in lactate levels increases the NADH/NAD^+^ ratio and decreases pyruvate dehydrogenase activity, inducing a reduction in total ATP production. Moreover, taurine deficiency leads to reduced fatty acid metabolism, downregulating the expression of peroxisome proliferator-activated receptor alpha (PPARα), an important nuclear receptor protein that promotes the fatty acid β-oxidation (Wen et al., 2019). These results highlight the essential role of taurine in the effective maintenance of cellular energy processes.

On the other hand, taurine works as a gene and transcription factor regulator in different models, including human hepatoma cells HepG2 (Park et al., 2006) and rodent heart (Schaffer et al., 2016). Genes regulated by taurine are involved in amino acid metabolism and protein synthesis. For instance, taurine depletion can provoke abnormal protein folding, thereby affecting longevity and cellular senescence (Ito et al., 2014). Cell injury and mitochondrial oxidative stress causes an imbalance between degradation and biosynthesis/folding of proteins, leading to the accumulation of unfolded or misfolded proteins, activating the unfolded protein response pathway (UPR) (Gharibani et al., 2015; Jong et al., 2015). By suppressing the UPR, taurine diminishes protein degradation, activation of chaperones, autophagy, and apoptosis that attenuates endoplasmic reticulum (ER) stress (Gharibani et al., 2013; Ito et al., 2015). This depicts taurine’s role in the protein quality control systems of the cells and its anti-senescence function.

After its uptake, hypotaurine is oxidized into taurine by the hypotaurine dehydrogenase (Vitvitsky et al., 2011). Extracellular taurine binds to GABA_A_ and glycine receptors augmenting its inhibitory effect (Jia et al., 2008). On the other hand, intracellular taurine concentrations are higher than extracellular levels, making it an organic osmolyte that contributes to the osmotic stress regulation in the cell (Oja and Saransaari, 2017). Moreover, taurine inhibits calcium release from internal stores, such as mitochondria, enhancing intracellular calcium modulation (Wu et al., 2005; Ramila et al., 2015). Other mechanisms described for taurine regulation of calcium influx include actions directed toward calbindin, calreticulin, and the Na^+^/Ca^2+^ exchanger in the outer cell membrane (Schaffer et al., 2002; Junyent et al., 2010).

Thus, due to its multiple physiological functions in cells, taurine has been proposed as a novel therapeutic agent for many human diseases including stroke, epilepsy, neurodegenerative diseases like Alzheimer’s disease (AD), retinal degeneration, heart failure, and mitochondrial diseases, such as mitochondrial encephalopathy with lactic acidosis and stroke-like episodes (MELAS), among others (Schaffer and Kim, 2018). Despite previous research regarding taurine’s effect in different tissues, its role in the nervous system, particularly the functional relationship with astrocytes, remains to be further elucidated. This aspect is important, as astrocytes are considered the main producers of taurine in the central nervous system (CNS) (Vitvitsky et al., 2011). Astrocytes also release taurine as a gliotransmitter, acting on other cells, mainly in neurons. Hence, a deeper understanding of taurine’s effect in astrocytes can contribute to a better insight of its physiological effects, which could potentially be used to ameliorate the course of several nervous system pathologies. This review, therefore, will provide an overview of the significant relationship between taurine and astrocytes, as well as its homeostatic and neuroprotective role in the pathologies of the nervous system.

## Taurine and Brain

Taurine is one of the most abundant amino acids in the human brain, although, its concentration declines with age, with decreasing values that range from 4–20 μmol/g during development to 1–9 μmol/g at adulthood (Wójcik et al., 2010; Roysommuti and Wyss, 2015). A study in adult male Wistar rats identified heterogeneous concentrations of taurine among different brain regions, showing higher levels in the pyriform cortex, caudate-putamen, cerebellum, and supraoptic nucleus, and lower concentrations in the midbrain reticular formation (Palkovits et al., 1986). During the rat’s postnatal growth, there is an increase in GABA, taurine and hypotaurine levels, which progressively decline until adulthood, in which its concentration reaches a plateau (Kontro et al., 1984). Moreover, a study in aged and young Wistar rats evidenced a significant decrease in the levels of taurine in the cerebellum, cortex, nucleus accumbens, and striatum of the aged animals (Benedetti et al., 1991). This suggests that taurine is required for an optimal postnatal brain development, which is supported by previous research evidencing disturbed maturation and migration of neurons, and a decreased number of astrocytes, in cat and monkey’s brains with taurine deficiency (Sturman, 1992; Pasantes-Morales, 2017).

### Taurine Synthesis and Transport

**FIGURE 1:**
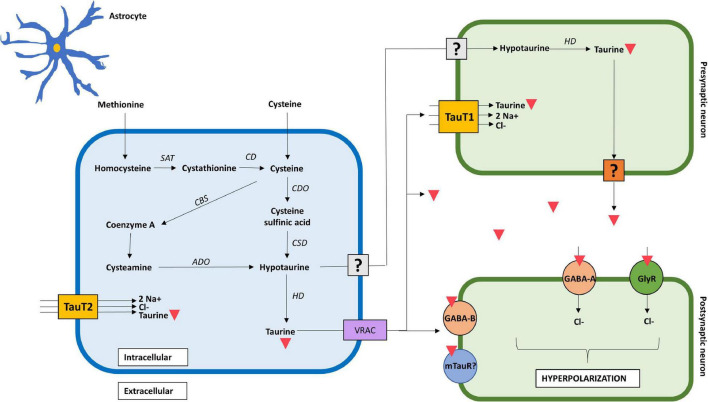
Mechanistic action of taurine in the brain. Taurine is synthesized in astrocytes from extracellular methionine and cysteine through three distinct pathways. It is exported as hypotaurine or taurine into the extracellular space; however, the mechanism in which hypotaurine exits the astrocyte and enters the neuron remains uncertain. Transport of taurine depends on TauT1 and TauT2 transporters, for neurons and astrocytes, respectively. The mechanism in which taurine exits the pre-synaptic neuron remains to be confirmed. Taurine acts as a full agonist for GABA_A_, GABA_B_, and glycine receptors in the post-synaptic neuron, increasing chloride conductance, and thus, hyperpolarizing the cell. The presence of a post-synaptic taurine receptor remains controversial. Taurine is represented by an inverted red triangle. ADO, aminoethanethiol dioxygenase; CBS, 2-cystathionine ß-synthase; CD, cysteine desulfurase/γ-cystathionase; CDO, cysteine dioxygenase; CSD, cysteine sulfinate decarboxylase; GlyR, glycine receptor; HD, hypotaurine dehydrogenase; TauT1, taurine transporter type 1; TauT2, taurine transporter type 2; SAT, S-adenosyl transferase; uncertain receptor/transporter–represented by a question mark.

Synthesis of taurine in the brain follows a similar enzymatic pathway compared with other tissues, such as muscle, adipose tissue and liver, however, the rate of production differs from one to another (Roysommuti and Wyss, 2015). This explains the slight differences in taurine concentrations between the brain and other tissues, reporting 9 μmol/g in the adult brain compared to 6 μmol/g in heart, 5 μmol/g in skeletal muscle, 2 μmol/g in the liver, and up to 40 μmol/g in retina (Wójcik et al., 2010). Three main pathways have been described for taurine synthesis in the brain (Hayes and Sturman, 1981). The first, and most common, depends on cysteine as its primary substrate, which is oxidized by cysteine dioxygenase to form cysteine sulfinic acid (CAD), and finally transformed to hypotaurine by cysteine sulfinic acid decarboxylase (CSAD) (Pasantes-Morales, 2017). The second pathway uses extracellular methionine as primary substrate, which undergoes different enzymatic reactions to form cysteine, which then follows the first pathway. The third pathway consists in the degradation of coenzyme-A yielding cysteamine, which is later transformed to hypotaurine by 2-aminoethanethiol dioxygenase (Banerjee et al., 2008). All three pathways form hypotaurine, which can be either transformed into taurine and released as such, or be released as hypotaurine and undergo its conversion to taurine in another cell, such as neurons (Roysommuti and Wyss, 2015; Figure 1).

Regarding neural cells, taurine predominates in astrocytes and neurons, demonstrated by the presence of specific enzymatic machinery needed for its synthesis (Ripps and Shen, 2012). Moreover, a metabolic coupling between astrocytes and neurons has been reported, in which astrocytes provide neurons with hypotaurine as a substrate for taurine production (Vitvitsky et al., 2011). Given the fact that neurons lack CSAD (Tappaz et al., 1994), they are unable to synthetize hypotaurine from cysteine, and thus, rely on astrocytic hypotaurine supply (Brand et al., 1997). A possible rationale behind the absence of a complete taurine synthetic pathway in neurons is that it spares cysteine for glutathione synthesis, prioritizing sulfur use for ROS buffering and xenobiotic detoxification in neurons (Banerjee et al., 2008). This suggests that *de novo* synthesis of taurine occurs predominantly in astrocytes, whereas neuronal concentration of taurine mostly depends on extracellular hypotaurine provided by astrocytes, and its conversion to taurine through intracellular enzymatic and non-enzymatic reactions. Furthermore, this explains why there is a lower concentration of both hypotaurine and taurine in neurons compared to that in astrocytes (Vitvitsky et al., 2011).

The cells in the brain can synthetize taurine from cysteine or methionine as mentioned above (Peck and Awapara, 1967), or import it *via* sodium and chloride dependent transporters TauT1 and TauT2 (Banerjee et al., 2008). TauT1 transporters predominate in cerebellar Purkinje cells and in bipolar cells in the retina, whereas TauT2 is associated with astrocytes and CA1 pyramidal cells in the hippocampus (Pow et al., 2002). In terms of regulation, Kang et al. (2002) used an *in vitro* BBB model to demonstrate TauT transporter upregulation in response to cellular damage, osmolality and declining taurine concentrations. Other factors such as hyperglycemia and oxidative stress also contribute to the TauT transporter regulation (Baliou et al., 2020).

### Taurine Release, Degradation, and Effects on Cellular Receptors

Taurine has a high transcellular gradient, with larger intracellular than extracellular concentrations, making it susceptible to changes in ionic concentrations (Oja and Saransaari, 2017). Furthermore, taurine’s molecular characteristics makes it more hydrophilic and less capable of trespassing biological membranes due to its sulfonyl group, resulting in a slower spontaneous efflux (Saransaari and Oja, 1992). Therefore, taurine release from the nerve terminal depends mostly on neuronal depolarization (Kamisaki et al., 1996). This is supported by a study by Saransaari and Oja (1991), which demonstrated taurine release by activation of *N*-methyl-D-aspartic acid (NMDA) and kainate receptors in a rat model. Nonetheless, the existence of taurine synaptic vesicles has not been completely confirmed, therefore it is suspected that its release relies on calcium-independent mechanisms, including volume-sensitive organic anion channels and TauT reverse transport (Kilb and Fukuda, 2017).

**FIGURE 2:**
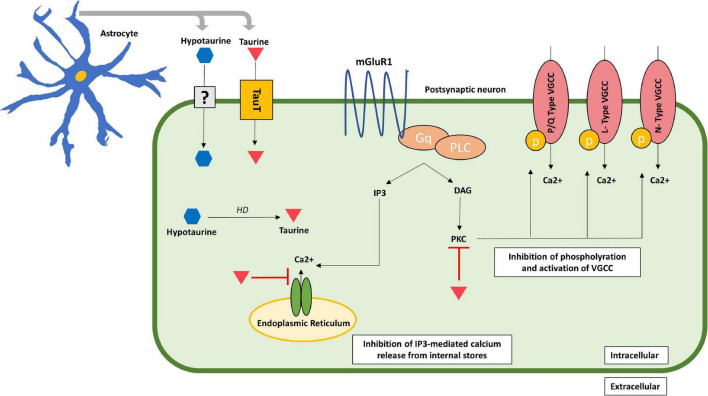
Acute neuroprotective effects of taurine in the post-synaptic neuron. Taurine inhibits calcium influx by inhibiting the IP3-mediated calcium release from internal storages and blocking PKC activity, which inhibits phosphorylation and activation of VGCC consequently decreasing calcium entry through these channels. Taurine is represented by an inverted red triangle, while hypotaurine is represented by a blue hexagon. DAG, diacylglycerol; IP3, inositol trisphosphate; mGluR1, metabotropic glutamate receptor type 1; PLC, phospholipase C; PKC, protein kinase C; TauT, taurine transporter; uncertain receptor/transporter–represented by a question mark; VGCC, voltage-gated calcium channels.

The existence of a specific taurine receptor in the human brain remains a controversial matter. Some authors have not ruled out its existence (Wu et al., 1992; Jakaria et al., 2019), while other evidence still questions it (Ripps and Shen, 2012). Several experimental studies in animals have explored, though not confirmed, the presence of the receptor (Kudo et al., 1988; López-Colomé et al., 1991; Frosini et al., 2003). Other authors have further proposed inhibition of the taurine receptor by guanosine-5′-triphosphate (GTP) in a dose-dependent way, implying not only its existence, but also the type of receptor as a metabotropic taurine receptor (mTauR) coupled to inhibitory G-proteins (Wu and Prentice, 2010). Nonetheless, future research is needed in order to corroborate the existence or not of the receptor, not only in neurons but also in other cell types such as glial cells. Further research showed taurine’s inhibitory effect on the protein kinase C (PKC), thereby blocking the phosphorylation and activation of voltage-gated calcium channels (VGCC), and therefore, decreasing calcium influx (Ohno and Nishizuka, 2002). Some authors propose that mTauR, if present, may have a similar effect as GABA_B_ receptors, so that its activation by taurine may lead to the activation of coupled inhibitory G-proteins, such as Go/Gi, which result in the inhibition of L-, N-, and P/Q-type VGCC (Leon et al., 2009; Traynelis et al., 2010; Wu and Prentice, 2010). Thus, the activation of mTauR and inhibitory G-proteins, leads to inhibition of phospholipase C (PLC), reduction in IP3 levels and consequently, inhibition of IP3-mediated release of Ca^2+^ from the internal storage pools (Leon et al., 2009; Traynelis et al., 2010; Wu and Prentice, 2010; Figure 2).

Taurine resembles the structure of the neurotransmitter GABA (Kletke et al., 2013; Ochoa-de la Paz et al., 2019). Such resemblance explains taurine’s ability to bind to GABA_A_ receptor (GABA_A_R), presumably at the interface of α/β subunits binding site. Moreover, taurine has been proposed as an endogenous agonist of the GABA_B_ receptors (GABA_B_R) in adult mouse brains (Albrecht and Schousboe, 2005). The same study also evidenced, through immunocytochemical analysis, that GABA_B_R, to which taurine binds, is located extrasynaptically. Taurine has been shown to activate both GABA_A_ and glycine receptors (GlyR) at a 0.1 mM concentration (Song et al., 2012). Despite this, Wu et al. (2008) evidenced in adult rats that taurine’s activation of GABA_A_R and GlyR varies among brain regions and is concentration-dependent. These authors observed that taurine at moderate concentrations (0.3 mM) activated GlyR, whereas at high concentrations (3 mM), acted as a weak agonist to GABA_A_R in neurons within the *substantia gelatinosa*. One explanation for this phenomenon may be the structural variability of these receptors due to the broad variety of combinations among its five subunits (Sigel and Steinmann, 2012).

In X*enopus* oocytes research, a comparison of native and recombinant selected types of GABA_A_R, showed that taurine acts as a full agonist at α_1_β_3_ receptors and as a partial agonist at α_1_β_3_γ_2_ receptor and GlyR, which causes an increase in chloride influx into the cell, and thus hyperpolarizes the post-synaptic neuron (Kletke et al., 2013; Ahring et al., 2016). Therefore, taurine enhancement of post-synaptic neuronal inhibition represents one of its most important neuroprotective mechanisms against excitotoxicity. Furthermore, experiments on amyloid beta (Aβ) models of neurotoxicity were carried out in chicks and rats, demonstrating that picrotoxin, a GABA_A_ antagonist, blocked taurine’s neuroprotective inhibitory effect (Louzada et al., 2004; Paula-Lima et al., 2005). Similarly, it has been proposed that the influx of chloride caused by taurine’s agonist action over GABA_A_R and GlyR, counteracts glutamate-induced excitotoxicity (Paula-Lima et al., 2005). However, taurine does not interact directly with the binding site for glutamate or glycine of the NMDA receptor (Foos and Wu, 2002). Therefore, it is assumed that taurine effects on NMDA intracellular signaling are indirect.

Excitotoxicity induced by glutamate has been well established experimentally, both *in vitro* and *in vivo*, mainly in epilepsy and stroke models (Lai et al., 2014). Glutamate binds to NMDA receptor, which leads to significant increases in intracellular calcium loads and catabolic enzyme activities, triggering mitochondrial membrane depolarization, caspase activation, ROS production, apoptosis, and necrosis (Dong et al., 2009). *In vitro* experiments of rat cultured neurons showed that in the presence of taurine, glutamate failed to increase intracellular calcium levels, showing a reduction of more than 50% in the presence of 25 mM of taurine (Chen et al., 2001; Wu et al., 2009). Actions of taurine on neurons might be related to the Na^+^/Ca^2+^ exchanger, which is a bidirectional ion transporter that couples the Na^+^ in one direction with that of Ca^2+^ in the opposite direction, depending on the electrochemical gradient of Na^+^ across the membrane (Blaustein, 1988; Blaustein et al., 1991; Takuma et al., 1994). During depolarization, the exchanger adopts a reverse mode, which promotes calcium influx. Taurine acts inhibiting the Na^+^/Ca^2+^ exchanger through the following mechanisms: increasing the phospholipid *N*-methyltransferase activity over the exchanger, enhancing the calcium efflux from the cell, and increasing the intracellular calcium close to the exchanger (Schaffer et al., 2003, 2010). Leon et al. (2009) studied the presence of taurine in rat cultured neurons exposed to glutamate, identifying an increase in anti-apoptotic Bcl-2 together with a downregulation of Bax which works in favor of apoptosis. Taurine administration showed a 60% increase in the Bcl-2:Bax ratio, meaning an overall inhibition of programmed cell death (Elmore, 2007; Khodapasand et al., 2015; Dorstyn et al., 2018). Furthermore, taurine inhibits caspase-9 and calpain activity as part of its anti-apoptotic mechanisms (Leon et al., 2009).

After its release, taurine undergoes three different processes for its degradation including transamination, oxidation and oxygenation (Caspi et al., 2018). The process of transamination consists in the formation of L-alanine and sulfoacetaldehyde from taurine and pyruvate (Shimamoto and Berk, 1979). Oxidation involves the formation of isethionate (2-hydroxyethane sulfonate) from taurine (Kondo et al., 1971). The oxygenation process consists of the use of taurine as a sulfur source by the sulfoacetaldehyde acetyltransferase, and finally into acetyl-CoA by the phosphate acetyltransferase (Eichhorn et al., 1997).

Taurine has been proposed as a neurotransmitter in the mammalian CNS (Huxtable, 1989; Wu and Prentice, 2010; Ripps and Shen, 2012; Kumari et al., 2013; Oja and Saransaari, 2017). This proposal has been based on the fact that taurine can be produced and released by pre-synaptic neurons, and exerts effects on post-synaptic neurons. However, for a substance to be considered a neurotransmitter it must meet the following criteria: (i) its synthesizing enzyme is present in the neuron; (ii) it is released upon neuron depolarization; (iii) it acts on a post-synaptic receptor; (iv) it causes a biological response on the post-synaptic neuron; and (v) it has an inactivation process after its release (Purves et al., 2001). So far, taurine complies with all but one of the mentioned criteria to be considered a neurotransmitter, which is the confirmation of the presence of a taurine specific receptor on the post-synaptic neuron. Furthermore, the intracellular gradient of taurine (around 400) has an intermediate position between established neurotransmitters (around 2000) and non-neurotransmitter aminoacids (less than 100) (Lerma et al., 1986; Oja and Saransaari, 2017).

## Taurine in Astrocytes

The cysteine dependent pathway is believed to be the main pathway for taurine biosynthesis in the brain (Banerjee et al., 2008). The cellular localization of CSAD has yielded controversial results *in vivo*. Nevertheless, incorporation of the radioactive 35S from cysteine into taurine has confirmed the presence of CSAD in astrocytes (Vitvitsky et al., 2011), making them fully capable of synthesis and accumulation of taurine (Hertz, 1979). In contrast, low CSAD activity, if any, is found in neurons (Pasantes-Morales, 2017), making neurons dependent on astrocytes for provision of hypotaurine and/or taurine (Banerjee et al., 2008). When co-cultured with neurons in a rat model, astrocytic hypotaurine decreased and taurine levels increased, revealing the crosstalk between these cell types for regulation of taurine synthesis (Brand et al., 1997). Although astrocytes have the enzymatic machinery for taurine synthesis, taurine can also be transported into the astrocyte through TauT2 transporters from interstitial fluid (Pow et al., 2002; Junyent et al., 2011). TauT has 12 hydrophobic transmembrane domains with cytosolic N- and C- terminals, and for each taurine molecule to be passed across the membrane, two Na^+^ ions and one Cl^–^ ion are required as a cotransport mechanism (Han et al., 2006).

Taurine has long been known to have an osmoregulatory role in the mammalian brain (Oja and Saransaari, 2017). Its release from neurons and astrocytes is directly proportional to a decrease in osmolarity (Pasantes-Morales and Schousboe, 1997). It has been shown that the replacement of 50 mM sodium ions by potassium evokes taurine release from rat’s cerebellar astrocytes (Schousboe and Pasantes-Morales, 1989). The same study evidenced that taurine release in a hyperosmotic media is decreased from both cerebellar astrocytes and granule cells, even when 50 mM of potassium is added. This is supported by Vitvitsky et al. (2011), who demonstrated that exposure to hypertonic conditions increases taurine levels in astrocytes up to 14% in 48 h. Contrastingly, there are other release mechanisms described for taurine, namely, a dose-dependent taurine release mediated by glutamate and kainate in animal cultured cerebellar astrocytes (Dutton et al., 1991). Such different release mechanisms of taurine from astrocytes support an astrocytic high concentration gradient and high affinity transport systems.

Astrocytic taurine concentration can vary according to different neurotoxic stimuli. Morken et al. (2005), working in mice, reported that when exposed to methylmercury (MeHg), the concentration of taurine increased in astrocytic monocultures, whereas it decreased in neurons in co-cultures. This suggests that there is a compensatory increase in levels of taurine in astrocytes in response to toxic stimuli, depicting another important neuroprotective effect of astrocytic taurine. Taurine release in neurons and astrocytes share similarities, including a delayed onset of taurine release after stimulation, however, some level of discrepancy exists between them. For example, exposure to tetrodotoxin and dihydropyridines inhibit neuronal taurine release, whereas taurine release from astrocytes remains intact despite the presence of a sodium or calcium channel blocker, respectively (Dutton et al., 1991).

Different studies have shown that taurine is released from astrocytes through the volume-regulated anion channels (VRACs) (Choe et al., 2012; Schober et al., 2017). These channels, discovered in 2014, are structured as an hetero-hexamer complex formed by the leucine-rich repeat-containing 8 (LRRC8) proteins (the essential LRRC8A and complementary LRRC8B to E), which are also involved in the release of other metabolites like glutamate and aspartate (Qiu et al., 2014; Voss et al., 2014). For instance, a recent study in mice showed that cerebral ischemia increased neuronal LRRC8A-dependent VRAC activity in the hippocampus, suggesting that VRAC contributed to increased glutamatergic release during ischemic damage (Zhou et al., 2020). Importantly, VRAC channels mediate swelling-activated Cl^–^ currents during decreases in systemic osmolarity and have been extensively studied in astrocytes, neurons and other non-neuronal cells like pituicytes and retinal Müller cells (Choe et al., 2012; Qiu et al., 2014; Mongin, 2016; Netti et al., 2018). Furthermore, it has been shown that primary rat astrocytes express LRRC8A, and that knockdown of astrocytic LRRC8A expression inhibits swelling-activated release of taurine (Hyzinski-García et al., 2014; Mongin, 2016; Schober et al., 2017). Expression of LRRC8A has been observed in rat astrocytes from brain regions such as cortex and hippocampus (Hyzinski-García et al., 2014; Formaggio et al., 2019). Thus, VRAC, LRRC8A, and taurine, seem to play a critical role in astrocytic homeostasis. Moreover, previous studies have shown that the highest levels of taurine in the brain are found in cerebral cortex, hippocampus, caudate-putamen, cerebellum, and in hypothalamic supraoptic nucleus (Hussy et al., 1997, 2000; Hatton, 2002). VRAC and VRAC-like currents have been reported in mice and rats in several brain regions including cervical sympathetic ganglions, CA1 region of the hippocampus, cerebral cortex, hypothalamus, and cerebellum (Leaney et al., 1997; Patel et al., 1998; Inoue et al., 2005; Sato et al., 2011; Zhang et al., 2011). For example, in the study by Choe et al. (2012), whole-cell voltage clamp recording demonstrated the absence of VRACs in rat’s neurons from the supraoptic nucleus, while confirming its presence in cultured astrocytes from the same site. This suggests that astrocytes, in contrast to neurons, may be the main responsible cells for the VRAC-mediated taurine release in the brain. However, the relation of this channel with taurine in humans and in other neural cells such as oligodendrocytes, NG2 glia, or microglia, has not been completely clarified (Wang et al., 2022). Moreover, some reports have shown that taurine acts as a potent GlyR and GABA agonist, reducing the release of vasopressin and oxytocin in magnocellular neurons (Deleuze et al., 1998; Song and Hatton, 2003). In rats, these two hormones regulate taurine secretion from pituicytes, thus creating a loop of paracrine intercellular communication (Rosso et al., 2004). Regarding its effects as a GlyR agonist, it has been shown that taurine activates GlyR through binding with homomeric subunits αH1 and αH2 in both animal and human cells (De Saint Jan et al., 2001). Consequently, transport inhibition of endogenous amino acids, such as taurine, contributes to the preservation of tonic activity in glycine receptors as well as in inhibitory neurons in the hippocampus (Mori et al., 2002), suggesting that taurinergic gliotransmission enhances inhibition on neurons.

As previously mentioned, taurine is mainly produced and released by astrocytes in the CNS. Therefore, metabolic and physiological aspects of taurine depend on an adequate function of these glial cells. This includes the communication between astrocytes and other cells, in particular, neurons. As mentioned above, astrocytes release hypotaurine, which then becomes the precursor for neuronal taurine production (Vitvitsky et al., 2011). Astrocytic taurine acts on GABA and GlyR favoring inhibitory activity in neurons (Song et al., 2012; Ochoa-de la Paz et al., 2019). Furthermore, taurine helps to control intracellular calcium levels and reduces the risk of developing excitotoxicity and apoptosis (Leon et al., 2009; Wu et al., 2009). Although not tested in co-culture with astrocytes, rat neurons treated with taurine increased both the incidence of synapse formation and the efficacy of synaptic transmission (Mersman et al., 2020). This suggests astrocytes may be involved in taurine-related neuronal synaptogenesis, as astrocytes have been reported to play a central role in synapse formation, function, and elimination (Chung et al., 2015). However, taurine metabolic coupling between astrocytes and neurons may be affected by external factors. For instance, *in vitro* treatment with manganese induced an increase in astrocytic taurine and a decrease in neuronal and astrocytic-neuronal co-culture taurine (Zwingmann et al., 2003). Another *in vitro* study in rat astrocytes, reported that rapid taurine accumulation is enhanced by hyperosmotic conditions (Beetsch and Olson, 1998). Despite these observations, it is still not clear whether other important coupling functions between astrocytes and neurons, such as the astrocyte-neuron lactate shuttle, or purinergic signaling, are affected by taurine.

Despite the controversy regarding taurine’s condition as a neurotransmitter, its categorization as a gliotransmitter can be clearly elucidated. For instance, for a substance to be considered an astrocyte gliotransmitter it must comply with the following criteria: (i) be synthesized and/or stored in astrocytes, (ii) have a physiologically triggered release, (iii) activate rapid responses in adjacent cells, and (iv) influence physiological processes (Volterra and Meldolesi, 2005). Other authors have proposed that substances that act as gliotransmitters can be released by calcium-independent non-exocytotic ways, as happens with taurine (Malarkey and Parpura, 2008; Hussaini and Jang, 2018). Taurine has proven to be synthesized and released from astrocytes, execute an inhibitory effect on neurons and influence multiple physiological processes. Therefore, taurine’s status as a gliotransmitter can be confirmed. However, whether its main effect is directly over the pre-synaptic or post-synaptic neuron, remains to be established.

Other aspects merit further exploration such as if any difference in taurine takes place in the functional regions of the astrocytes (i.e., endfeet vs. soma), in astrocytic subtypes (i.e., protoplasmatic gray matter vs. fibrous white matter astrocytes), and if taurine is somehow involved in the function of glymphatic system.

## Neuroprotective Effects of Taurine Released From Astrocytes

As mentioned above, taurine acts as a gliotransmitter with a neuroprotective role in the brain. When released from astrocytes, it can either bind to neuronal post-synaptic receptors such as GABA_A_R and GlyR (Wu and Prentice, 2010), or enter the neuron *via* TauT1 and TauT2 transport proteins where it triggers intracellular signaling pathways. However, taurine’s overall inhibitory effects combine both extracellular and intracellular processes.

Regarding the extracellular effects of taurine, it involves binding to neuronal GABA_A_R and GlyR, augmenting chloride conductance, thereby hyperpolarizing the cell and neutralizing NMDA-mediated glutamate excitotoxicity (Wu and Prentice, 2010). Furthermore, taurine release from the cells decreases Ca^2+^ entry by limiting the available Na^+^ needed for the adequate functioning of the Na^+^/Ca^2+^ exchanger in the cell membrane (Schaffer and Kim, 2018).

On the other hand, intracellular effects of taurine released from astrocytes involve direct and indirect actions on calcium homeostatic pathways in neurons. By reducing calcium overload, taurine prevents excitotoxicity, mitochondrial stress, and activation of apoptotic pathways (Junyent et al., 2010). Studies with cultured neurons treated with glutamate showed a significant increase in intracellular calcium levels, however, when taurine was added, such calcium elevation returned to near basal levels (Foos and Wu, 2002). As glutamate binds to its metabotropic receptor, IP3 increases, as does IP3-mediated calcium release from internal storages. However, when taurine was added, there was no increase in IP3 nor in intracellular calcium levels (Foos and Wu, 2002). This suggests taurine’s neuroprotective effect toward glutamate-induced calcium overload and consequent excitotoxicity. Furthermore, authors have proposed that taurine activates coupled inhibitory G-proteins, which in turn, inhibit phosphorylation of VGCC, thus preventing its activation induced by glutamate (Wu and Prentice, 2010). Therefore, by inhibiting calcium influx from VGCC, taurine provides another mechanism for regulation of intracellular calcium concentrations.

**FIGURE 3:**
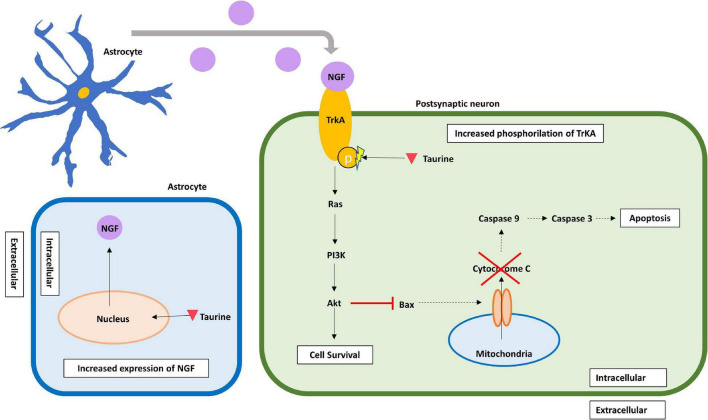
Delayed neuroprotective effects of taurine in the post-synaptic neuron. Taurine increases the expression of NGF, presumably in astrocytes from where it is released. Additionally, intracellular taurine increases phosphorylation of TrkA, a specific receptor for NGF, thus enhancing the NGF/Akt/Bad anti-apoptotic pathway. By activating this pathway, taurine enhances Bax inhibition, which blocks Cytochrome C release from mitochondria, and inhibits caspase-mediated apoptotic pathways. Taurine is represented by an inverted red triangle, while NGF is represented by a magenta circle. HD, hypotaurine dehydrogenase; NGF, nerve growth factor; PI3K, phosphatidylinositol 3-kinase; TrkA, tropomyosin receptor kinase A.

In a type II diabetes mellitus rat model, intracellular taurine has proven to inhibit neuronal apoptosis in the brain, more specifically in the hippocampal CA1 pyramidal cells by acting on the NGF/Akt/Bad pathway (Wu et al., 2020). The addition of taurine to rat’s hippocampal cultured neurons increased the expression of nerve growth factor (NGF) and augmented phosphorylation of the NGF receptor, tyrosine kinase receptor type A (TrkA), activating an important anti-apoptotic pathway. Furthermore, hippocampal rat neurons treated with taurine showed a decreased expression of Bax, a diminished release of cytochrome C and a reduction in caspase-3 and caspase-8 activity, thereby contributing to neuronal survival by inhibiting apoptotic signaling pathways (Li et al., 2017). Finally, some evidence has emerged suggesting that taurine concentration may be related with the development of Parkinson’s disease (PD) (Zhang et al., 2016; Che et al., 2018). For example, a study with 110 PD treated and untreated patients, found that PD patients exhibited lower levels of plasma taurine than control individuals (51.01 ± 29.07 vs. 133.83 ± 45.91 μmol/L, *p* < 0.001) (Zhang et al., 2016). Moreover, in a mice PD model, using primary neuron-glia cultures, it was found that taurine administration (150 mg/kg), attenuated the aggregation of α-synuclein, decreased the inflammatory response by microglia and provided dopaminergic neuroprotection (Che et al., 2018). Furthermore, a study in C57Bl/6j male mice showed that taurine administration significantly decreased the total number of ionized calcium-binding adapter molecule 1 (Iba1)-expressing microglia in the dentate gyrus supporting its anti-inflammatory role (Gebara et al., 2015). These combined results suggest the importance of taurine neuroprotection in neurodegenerative diseases such as PD. Moreover, as astrocytes are the main taurine releasing cells in the CNS, we suggest most of these neuroprotective effects may be attributed to astrocytes (Figure 3).

## Taurine as a Therapeutic Alternative in Nervous System Pathologies

Taurine’s neuroprotective role in neurons and astrocytes represent a novel potential therapeutic approach toward several nervous system diseases. By augmenting neuronal inhibition mediated by GABA_A_R and GlyR, taurine has proven to enhance neuronal inhibition, which remains useful in diseases such as epilepsy, characterized by a hypersynchronous and hyperexcitable neuronal circuit. Winkler et al. (2019) identified that the addition of 0.5 μmol/L of taurine significantly increased the anticonvulsant effect of 100–200 μmol/L of pentobarbital in mice. Kainic acid administration is one of the most frequently used models to mimic human temporal lobe epilepsy in animals. Either subcutaneous or intraperitoneal injection of taurine, preceding kainic acid administration, resulted in seizure prevention and significant reduction in neuronal cell death in the hippocampus of male mice (El Idrissi et al., 2003; Junyent et al., 2009). This suggests that taurine’s action as a positive modulator of GABA_A_R could be considered as part of epilepsy therapy. In humans with epilepsy, including in children (Fukuyama and Ochiai, 1982), the effects of taurine oral administration on seizures has been examined (as reviewed by Oja and Saransaari, 2013). Most of these studies were conducted in the 70’s and 80’s, and showed a wide variation in the results. For instance, some reported disappearance of seizures in patients with intractable epilepsy, while others failed to show any improvement, despite comparable dosages. However, these clinical trials had many issues, such as a small number of participants, diverse range of epilepsy types and no control groups, among others. According to the webpage, clinicaltrials.gov, no clinical trial examining effects of taurine on epilepsy is currently ongoing. Therefore, novel and much better designed clinical trials examining taurine effects on epilepsy should be proposed in the future. On the other hand, taurine’s role in the inhibition of neuronal apoptotic pathways and attenuation of oxidative stress, could represent a potential adjuvant therapy, particularly in neurodegenerative diseases such as AD. It has been shown that small concentrations of taurine in neuronal cultures from chick embryo retinas can decrease Aβ peptide aggregation and the underlying neuronal death (Louzada et al., 2004). In APP/PS1 AD transgenic mice, taurine administration was shown to bind oligomeric Aβ peptides and improve cognition (Jang et al., 2017). Moreover, decreased taurine levels, as detected by ion exchange in the temporal cortices of patients with AD could lead to higher aggregation and more rapid disease progression, highlighting the potential role of taurine as co-adjuvant in the delaying of neurodegenerative processes (Arai et al., 1985; Chen et al., 2019).

Taurine’s neuroinflammatory modulation represents another viable mechanism in which it can be implemented in therapeutic approaches toward diseases such as traumatic brain injury and ischemic stroke, characterized by enhanced neuroinflammatory processes. By decreasing the expression of proinflammatory cytokines through the inactivation of microglia-mediated NOX2-NF-κB cascade, taurine may contribute to the amelioration of inflammatory changes (Che et al., 2018; Bhat et al., 2020; Tian et al., 2020). For example, administration of high doses of taurine in a rat model of intracerebral hemorrhage resulted in inactivation of proinflammatory pathways, thereby limiting neuronal damage and white matter injury, accompanied by a decrease in neutrophil infiltration and glial activation (Zhao et al., 2018). Furthermore, in an *in vitro* model for ischemic stroke, 88% of neurons pretreated with taurine survived after being exposed to high levels of glutamate (Prentice et al., 2017). Similarly, in the same study, the administration of taurine in an *in vivo* rat stroke model resulted in the inhibition of components within the ER stress pathway, reducing neuronal glutamate excitotoxicity. Therefore, taurine could have a beneficial effect in neuroinflammatory diseases and ischemic or traumatic brain injury.

The development of biological markers, or biomarkers, for nervous system diseases is actually one of the most important research topics in the field of neuroscience. The interest in the development of biomarkers is based on several current challenges these group of diseases pose, such as the lack of comprehension of the pathological mechanism, together with difficulties in the prevention, in particular early interventions, confirmatory diagnosis and treatment. According to the most accepted definition, a biomarker should be objectively measured or evaluated, and must be able to indicate (and differentiate) physiological activity from pathological processes or from pharmacological responses (Strimbu and Tavel, 2010). In addition, ideal biomarkers should benefit from being less invasive and fast to measure, providing results in minutes or hours, rather than days or weeks. Therefore, many biomarkers are explored in blood, saliva, or urine samples, as well as in imaging techniques including magnetic resonance or positron emission tomography (PET) (Young et al., 2020; Zetterberg and Schott, 2022). In fact, live imaging of brain metabolism is currently proposed as an interesting biomarker method both in preclinical and clinical settings (Zhu et al., 2018). Therefore, as taurine plays and important role in brain metabolism, and is mainly produced in astrocytes (the principal homeostatic regulators in the SNC), taurine can be suggested as a possible brain biomarker.

Several techniques exist to determine the metabolic activity of the brain in living individuals. The most commonly used in humans are functional magnetic resonance imaging (fMRI), PET, magnetic resonance spectroscopy (MRS) and metabolomics in tissue samples (Barros et al., 2018). Taurine is one of the metabolites that can be determined through MRS thus possibly serving as a biomarker. For instance, taurine concentrations, determined through MRS, were measured in primitive neuroectodermal tumors (PNT) of pediatric patients, finding a significant increase of this metabolite, which helped to differentiate PNT from other tumors (Kovanlikaya et al., 2005). Metabolic profiling of taurine has also been extended to other neural derived tumors such as medulloblastoma, neuroblastoma, and retinoblastoma (Kohe et al., 2018). Recently, a brain MRS study in cannabinoid users, found that taurine concentration was directly correlated with glutamate, and with the frequency of cannabinoid use (Newman et al., 2022). Furthermore, MRS measurements determined elevated concentrations of taurine in the cerebellar vermis of bipolar patients (Magnotta et al., 2022), and levels of taurine were significantly related to the duration of illness in schizophrenic patients (Shirayama et al., 2010). Hence, taurine MRS measurements can be applied to a large extent of neurological and neuropsychiatric diseases.

Taurine values have also been examined in biological samples such as blood and cerebrospinal fluid (CSF). A recent study found that taurine plasma levels correlated with the strength-duration time constant, an axonal excitability indicator, established to predict survival in amyotrophic lateral sclerosis (ALS) (Nakazato et al., 2022). Furthermore, taurine was selected as a biomarker metabolite that helps to distinguish between patients with amnestic mild cognitive impairment and healthy controls (Sun et al., 2020). Plasmatic taurine has also been used as a predictor of poor outcome in patients with subarachnoid hemorrhage, finding that those patients with a sixfold increase in taurine at admission ended with a poor outcome, compared with those with a favorable outcome who had only a twofold increase (Barges-Coll et al., 2013). Similar findings were observed in the CSF of severe brain-injured patients, where taurine was significantly increased in subdural or epidural hematomas, contusions and generalized brain edema (Stover et al., 1999). Elevated concentrations of taurine in plasma have also been reported in psychiatric diseases, such as depression (Altamura et al., 1995). Similar findings have been observed in preclinical models. Song et al. (2021) studied a rat model treated with 20 mg/kg of fluoxetine daily, evidencing that repeated doses of fluoxetine, a selective serotonin reuptake inhibitor, led to decreased concentrations of taurine and other astrocytic metabolites in the medial prefrontal cortex. Moreover, Wu et al. (2017) reported that taurine pre-administration in a rat depression model counteracts the rise in glutamate and inflammatory mediators such as corticosterone. Furthermore, in the same study, the administration of taurine prevented the reduction in substances such as 5-hydroxytryptamine (5-HT), dopamine, noradrenaline, and even upregulated the expression of neurotrophic factors like fibroblast growth factor-2 (FGF-2), vascular endothelial growth factor (VEGF), and brain derived neurotrophic factor (BDNF), that tend to fall under stress conditions. This suggests taurine’s compensatory effects in response to stress-induced depression, demonstrating that taurine’s antidepressant properties could possibly contribute to neuropsychiatric disease therapeutics.

An important question about the possible use of taurine as a therapeutic option in nervous system pathologies refers to the delivery mode in the organism. Oral delivery is the most common and easy method of taurine administration, and most trials in humans use this form of delivery. In fact, oral taurine administration, up to 4 grams in healthy adults or up to 6 grams daily for 6 months in children with fatty liver, has been given without toxic side effects (Obinata et al., 1996; Ghandforoush-Sattari et al., 2010). However, despite encouraging results in many preclinical works, some reports in animal studies have shown that oral administration is not as effective as taurine injection, and that prolonged consumption may even produce some negative effects in rats (Eppler et al., 1999; El Idrissi et al., 2003). Therefore, the need to explore additional and more efficient administration routes. For instance, intravenous delivery of taurine (up to five grams) has been given safely to patients during myocardial revascularization (Milei et al., 1992). However, intravenous delivery of taurine has not been tested in humans with neurological or neuropsychiatric diseases. In addition to oral and intravenous delivery, other systemic administration routes such as intraperitoneal injection may be implemented, although they tend to be more invasive and risky, increasing the possibility of bleeding or infection. Systemic administration is also limited by the BBB, which restricts taurine passage into CNS parenchyma. Furthermore, the BBB transport of taurine into the brain can be affected by the presence of cytokines such as tumor necrosis factor (TNF) (Kang et al., 2002; Lee and Kang, 2004). Therefore, novel methods that bypass the BBB should be tested. For instance, intranasal delivery of medications have been shown to be a practical and non-invasive method to evade the BBB and reach the CNS (Hanson and Frey, 2008). In mice, intranasal delivery of taurine produced anxiolytic effects in animals treated with strychnine, picrotoxin, yohimbine, or isoniazid (Jung and Kim, 2019). Another strategy may involve the use of taurine nanocarriers aimed at CNS cells. Recently, a functionalized nanoparticle with taurine and graphene oxide was designed, and tested successfully in rats (Pan et al., 2021).

## Conclusion

There is compelling evidence of taurine’s neuroprotective and homeostatic effects in the CNS, including maintenance of cellular energy processes, intracellular calcium modulation, osmotic stress regulation, and protection against glutamate-induced excitotoxicity, among others. The metabolic coupling for taurine synthesis and degradation between astrocytes and neurons, evidence neuronal dependence on astrocytes for the adequate functioning of the mentioned effects in the brain. Finally, it could be relevant to explore the effects of taurine in other glial cells such as microglia or oligodendrocytes, in functions such as antioxidative protection and demyelination and its association with astrocytes. Therefore, the astrocyte’s role in taurine-induced neuroprotective functions should be considered as a promising therapeutic target in the management of several neurodegenerative and neuropsychiatric diseases in the near future.

## Author Contributions

SR-G: literature review, writing the manuscript, and making the figures. SG-M, GM-R, and EO-G: literature review and writing the manuscript. RC-P: writing and editing the manuscript. RG-R: original draft preparation and writing and editing the manuscript. All authors contributed to the article and approved the submitted version.

## Conflict of Interest

The authors declare that the research was conducted in the absence of any commercial or financial relationships that could be construed as a potential conflict of interest.

## Publisher’s Note

All claims expressed in this article are solely those of the authors and do not necessarily represent those of their affiliated organizations, or those of the publisher, the editors and the reviewers. Any product that may be evaluated in this article, or claim that may be made by its manufacturer, is not guaranteed or endorsed by the publisher.
